# Sleep-Deprivation Induces Changes in GABA_B_ and mGlu Receptor Expression and Has Consequences for Synaptic Long-Term Depression

**DOI:** 10.1371/journal.pone.0024933

**Published:** 2011-09-27

**Authors:** Ramakrishna Tadavarty, Padmesh S. Rajput, Jennifer M. Wong, Ujendra Kumar, Bhagavatula R. Sastry

**Affiliations:** 1 Neuroscience Research Laboratory, Department of Anesthesiology, Pharmacology and Therapeutics, Faculty of Medicine, The University of British Columbia, Vancouver, British Columbia; 2 Faculty of Pharmaceutical Sciences, The University of British Columbia, Vancouver, British Columbia; Centre National de la Recherche Scientifique - , University of Bordeaux, France

## Abstract

Long term depression (LTD) in the CA1 region of the hippocampus, induced with a 20-Hz, 30 s tetanus to Schaffer collaterals, is enhanced in sleep-deprived (SD) rats. In the present study, we investigated the role of metabotropic glutamate receptors (mGluRs), γ-Aminobutyric acid (GABA) B receptors (GABA_B_-Rs) and N-methyl-D-aspartic acid receptors (NMDARs) in the LTD of the population excitatory postsynaptic potential (pEPSP). The requirement of Ca^2+^ from L- and T- type voltage-gated calcium channels (VGCCs) and intracellular stores was also studied. Results indicate that mGluRs, a release of Ca^2+^ from intracellular stores and GABA_B_-Rs are required for LTD. Interestingly, while mGlu1Rs seem to be involved in both short-term depression and LTD, mGlu5Rs appear to participate mostly in LTD. CGP 55845, a GABA_B_-R antagonist, partially suppressed LTD in normally sleeping (NS) rats, while completely blocking LTD in SD rats. Moreover, GS-39783, a positive allosteric modulator for GABA_B_-R, suppressed the pEPSP in SD, but not NS rats. Since both mGluRs and GABA_B_-Rs seem to be involved in the LTD, especially in SD rats, we examined if the receptor expression pattern and/or dimerization changed, using immunohistochemical, co-localization and co-immunoprecipitation techniques. Sleep-deprivation induced an increase in the expression of GABA_B_-R1 and mGlu1αR in the CA1 region of the hippocampus. In addition, co-localization and heterodimerization between mGlu1αR/GABA_B_-R1 and mGlu1αR/GABA_B_-R2 is enhanced in SD rats. Taken together, our findings present a novel form of LTD sensitive to the activation of mGluRs and GABA_B_-Rs, and reveal, for the first time, that sleep-deprivation induces alterations in the expression and dimerization of these receptors.

## Introduction

Sleep is imperative to majority of species across the animal kingdom. Although it is implicated in a variety of functional roles [1], the idea that sleep facilitates learning and consolidation of memories has attracted special attention in recent years. Memories, when initially acquired, exist in a fragile state, often vulnerable to external amnesic influences. At a later stage, in a process termed consolidation, labile memory traces are converted to more enduring and long-lasting forms [2]. Following recall, however, memories often become transient once again, requiring reconsolidation to regain the lost stability [3]. It is during the consolidation and reconsolidation stages, that sleep is considered critical. Any disturbance in the normal sleep pattern may therefore adversely affect learning and formation of memories. Supporting this notion, several behavioural studies have shown that sleep-deprivation post-training is detrimental to learning and compromises task re-performance [4]. A normal sleep, on the other hand, actively promotes and enhances the consolidation of declarative and procedural memories, resulting in a significant improvement in task re-performance [5]. At the cellular level, however, the effects of sleep on memory are not well understood.

In the central nervous system (CNS), synapses are considered to be the loci for memories. Activity-mediated forms of plasticity, long-term potentiation (LTP) and long-term depression (LTD), cause input-specific, long-lasting and reversible modifications to synapses [6], and hence are widely accepted as cellular processes crucial for learning and memory. If this is true, learning and memory impairments observed post sleep-deprivation at the behavioural level must reflect as alterations in plasticity at the synaptic level. In fact, several studies correlate sleep-deprivation induced cognitive deficits to a disruption in the induction and/or maintenance of LTP in the hippocampus and elsewhere in the CNS [7].

In addition to the above effects on LTP, we recently showed that sleep-deprivation results in an enhancement of LTD [8]. This finding is especially interesting because LTD is implicated in a variety of functional roles in the CNS. It protects neurons by dampening neuronal hyper-excitability, prevents a saturation of neuronal network activity [9], and facilitates the formation of certain types of memories [10]. In adult/aged rats, however, an enhanced susceptibility to LTD has been found to not only interrupt the maintenance of LTP [11], but also account for a slower pace of learning, poor retention and faster forgetting [12]. These observations support a vital role for LTD in the formation of memories, the modulation of LTP, and in normal functioning of the CNS.

At least two major forms of LTD, depending on the activation of metabotropic glutamate receptors (mGluRs) or N-methyl-D-aspartic acid (NMDA) receptors, have been proposed [13], [14]. mGluR- and NMDA receptor- mediated LTDs seem to vary significantly in their induction, expression and maintenance mechanisms, but require a postsynaptic elevation in [Ca^2+^]_i_ through discrete sources [15]. γ- Aminobutyric acid (GABA), mediates inhibition in the CNS, by activating two types of receptors: ionotropic GABA_A_/GABA_C_ receptors and metabotropic GABA_B_ receptors. These receptors are located strategically to regulate both “inputs-to” and “outputs-from” the pyramidal neurons, and therefore impart a powerful regulatory influence on their net-excitability [16]. Hence it is not surprising that alterations in the efficacy of the inhibitory transmission, owing to changes in receptor expression [17] or short-/long- term plasticity of the inhibitory postsynaptic currents (IPSCs) [18], [19], [20] significantly affect the induction of both LTP and LTD [21]. Hence, in the present study, we examined the involvement of NMDA receptors, mGluRs, Ca^2+^-release from intracellular stores and GABA_B_ receptors in LTD.

G-protein coupled receptors (GPCRs) were traditionally thought to exist and function in monomeric entities. However, accumulating biochemical and biophysical evidence indicates that most, if not all GPCRs assemble as homo- and/or heterodimers [22], [23]. While there is no consensus on the precise physiological role of such interactions, for some GPCRs like the GABA_B_ receptors, heterodimerization seems to be obligatory. In a GABA_B_-R1-GABA_B_-R2 complex, the GABA_B_-R1 fraction imparts sensitivity to endogenous ligands, whereas the GABA_B_-R2 subtype enables coupling of the receptor to G-proteins. Also, this association seems to be sufficient to overcome the endoplasmic reticulum (ER) retention signal for GABA_B_-R1 subtype, thereby allowing the receptor to traffic and express on the cell surface. Therefore, the co-expression of both subtypes seems to be necessary for a functional GABA_B_ receptor [24], [25], [26]. Whether GABA_B_-R1s dimerize with other closely related family 3 GPCRs, such as mGluRs, is presently unclear. However, since GABA_B_Rs and mGlu1Rs are both localized in the perisynaptic regions of the dendritic spines/shafts [27], [28], a physical interaction between these receptors is an interesting possibility [29]. The resulting interaction may change the basic function of either receptor, such that the activation of one receptor produces a synergistic/antagonistic effect on the function of the other with significant implications for synaptic plasticity. In the current study, we, therefore, examined if GABA_B_-R1 and mGlu1αR heterodimers exist in hippocampus and whether they are changed during sleep-deprivation using a combination of immunohistochemistry, western blot analysis and co-immunoprecipitation techniques.

Results from our combined electrophysiological, morphological and biochemical approach indicate that, a) 20-Hz input stimulation-induced LTD requires the activation of mGluRs & GABA_B_Rs and a release of Ca^2+^ from intracellular stores, b) the expression of mGlu1αR and GABA_B_-R1 is significantly increased in SD rats, c) co-localization and heterodimerization between mGlu1αR & GABA_B_-R1 and mGlu1αR & GABA_B_-R2s is enhanced in SD rats. These findings, to our knowledge, provide the first evidence for specific changes in GABA_B_- & mGlu1α- receptor expression and possible dimerization during sleep-deprivation.

## Materials and Methods

### Animals

3–4 week old male Wistar rats were purchased from the Animal Care Centre, The University of British Columbia. All experiments were performed in accordance to the guidelines of the Canadian Council on Animal Care and the University of British Columbia committee on Animal Care (Protocol #A07-0536).

### Sleep deprivation

Animals were total-sleep-deprived by gentle-handling. For 12 h during the light period, animals were kept under constant observation of the experimenter and kept awake by mild tapping of the cage, gentle prodding with a brush, etc., when they assumed a sleeping posture. Our procedure, unlike other methods used in previous studies [30], [31], [32], did not induce behavioural stress in the animals and no significant difference was found in the serum-corticosterone levels between normally sleeping and SD rats [8].

### Brain-slice preparation

Briefly, animals were anaesthetized with halothane and decapitated using a guillotine at the end of the 12 h light period. Brains were then rapidly removed and transverse sections of the hippocampus (400 µm) obtained by procedures routinely used in our laboratory [20]. Slices were cut in ice cold sucrose solution containing (in mM): 234 sucrose, 2.5 KCl, 28 NaHCO_3_, 1.25 NaH_2_PO_4_, 3 pyruvic acid, 1 ascorbic acid, 7 MgCl_2_, 0.5 CaCl_2_ and 10 dextrose (saturated with 95% O_2_/5% CO_2_); pH was adjusted to 7.35–7.4 with NaOH. The CA3 region was cut off from the slice to diminish the influence of spontaneous activity from CA3 neurons. Slices were then kept in an incubation chamber filled with artificial cerebrospinal fluid (ACSF) containing (in mM): 120 NaCl, 3 KCl, 1.8 NaH_2_PO_4_, 26 NaHCO_3_, 2 CaCl_2_, 2 MgCl_2_ and 10 dextrose (saturated with 95% O_2_/5% CO_2_); pH was adjusted to 7.35–7.4 with NaOH, for 1–1.5 h at 25–26°C. Following incubation, individual slices were transferred into a recording chamber superfused with ACSF at a rate of 1.5–2 ml/min.

### Electrophysiological procedures

pEPSPs were evoked with a bipolar platinum stimulating electrode placed in the stratum radiatum of the CA1 region of hippocampus and recorded from the apical dendrites using a recording glass micropipette (filled with ACSF). Control stimulation frequency was set at 0.05 Hz with square pulses (0.1–0.2 ms duration). After stable responses were obtained, an input-output curve was constructed and the stimulus intensity high enough to evoke a half-maximal pEPSP was chosen. This was done to allow enough room for facilitation and/or depression without contamination from population spikes. After a 10–15 min pre-tetanic control recording, LTD was induced using a tetanic stimulation (20-Hz, 30 s) as described in Tadavarty et al., 2009 [8].

### Immunohistochemistry

Immunohistochemical studies were performed on coronal sections of rat brains, as described previously [33]. Briefly, normally sleeping or SD rats were anaesthetized by an intraperitoneal injection of sodium pentobarbital, and the brains fixed by transcardial perfusion with 0.9% cold heparinized saline and 4% paraformaldehyde. Post-fixation, the brains were taken out and cryoprotected in 20% sucrose and 40% sucrose solution. Following a wash in cold water, 40 µm thick sections were prepared using the Leica 1200 s vibratome. Free-floating hippocampal sections were incubated in 1% H_2_O_2_ (for 15 min) and 0.2% Triton X-100 (for 15 min), washed 3× in TBS post-incubation in between each treatment. The sections were then blocked in 5% normal goat serum (NGS; for 1 h at RT), and incubated with primary antibodies, specific to mGlu1αR (BD Biosciences, Pharmingen, San Diego, CA, USA), GABA_B_-R1 (Santa Cruz Biotechnology, Santa Cruz, CA) and GABA_B_-R2 (Santa Cruz Biotechnology, Santa Cruz, CA) at 1∶300 dilution in 1% NGS, overnight at 4°C in a humid atmosphere. Following three subsequent washes in TBS, sections were incubated for 1 h with biotinylated secondary antibodies. The avidin-biotin complex method was used to detect the antigen (ABC kit, Vector laboratories, Burlingame, California) and 3, 3′-diaminobenzidine tetrachloride (DAB, 0.2 mg/ml) containing 0.001% H_2_O_2_ was used to visualize the reaction. Sections were then mounted on slides, viewed and photographed using the IBRE microscope equipped with a Cool Snap camera.

### Indirect immunofluorescence

Co-localization of mGlu1αR with GABA_B_-R1/R2 and GABA_B_-R1 with GABA_B_-R2 was studied in normally sleeping and SD rats, as described previously [34]. Briefly, brain sections passing through hippocampus were selected and incubated in 0.2% Triton X-100 (for 15 min) and washed 3× with TBS for 10 min. The sections were then blocked in 5% NGS for 1 h at RT, and incubated overnight at 4°C in a humid atmosphere with primary antibodies (at 1∶300 dilution in 1% NGS) in the following combination: mouse anti-mGlu1αR and rabbit anti-GABA_B_-R1 and GABA_B_-R2; guinea pig anti-GABA_B_-R1 and rabbit anti-GABA_B_-R2. This was followed by incubation with mixtures of Alexa 594 (red) and Alexa 488 (green)-conjugated goat anti-mouse or goat anti-rabbit secondary antibodies. Finally, the sections were mounted on slides, viewed and photographed using the Leica DMLB microscope equipped with a Retiga 2000R camera.

### Western blot and Co-Immunoprecipitation

Western blot and co-immunoprecipitation were performed on the tissue lysate prepared from the hippocampus of normally sleeping and SD rat brains, as described previously [34]. Briefly, the hippocampal tissue lysate was solubilized in a homogenizing buffer containing (in mM): 62.5 Tris-HCl, 50 dithiothretiol (DTT), 2% SDS, and 10% glycerol. Protein concentration in the tissue was estimated using the Bradford protein assay. 20 µg of protein was then solubilized in Laemmli buffer with 5% 2-mercaptoethanol and heated at 99°C for 5 min. Samples were then fractionated by electrophoresis on a 7% SDS polyacrylamide gel and then transferred onto a nitrocellulose membrane. The membrane was blocked with 5% non-fat dry milk for 1 h at RT and subsequently incubated overnight at 4°C with primary antibodies (at 1∶500 dilutions in 5% bovine serum albumin) specific to mGlu1αR, GABA_B_-R1 and GABA_B_-R2. Membranes were then incubated with goat anti-mouse or goat anti-rabbit secondary antibodies for 1 h at RT. Bands were detected with a chemiluminescence reagent and images were taken using the Alpha Innotech Fluorchem 800 (Alpha Innotech Co., San Leandro, CA) gel box imager. β-actin was used as the house-keeping protein.

For co-immunoprecipitation experiments, tissue lysates prepared from normally sleeping and SD rats were centrifuged and the pellet was further solubilised in 1 ml of radio-immunoprecipitation assay (RIPA buffer; containing, 150 mM NaCl, 50 mM Tris–HCl, 1% Nonidet P-40, 0.1% SDS, and 0.5% sodium deoxycholate, pH 8.0), for 1 h at 4°C. Tissue lysates were then incubated with monoclonal mGlu1αR or polyclonal GABA_B_-R1 antibodies (at 1∶500 dilutions) overnight at 4°C on a rocking shaker. 25 µl of protein A/G-agarose beads were added to each tube to immunoprecipitate antibody for 2 h at 4°C. Beads were then washed three times in RIPA buffer and solubilized in Laemmli sample buffer (Bio-Rad) containing 5% β-mercaptoethanol. The samples were heated at 99°C for 5 min before being fractionated by electrophoresis on a 7% SDS-polyacrylamide gel. The fractionated proteins were transferred to a 0.2 µM nitrocellulose membrane in transfer buffer. Membranes were blotted with antibodies specific to GABA_B_-R1 (Millipore Corporation, Billerica, MA) and GABA_B_-R2, as described in the western blot analysis section.

### Data acquisition and Statistical analysis

Synaptic potentials were recorded using the Axopatch 200A (Axon Instruments) amplifier connected to a Digidata 1220 interface. Low pass filtering was set at 5 kHz. Recordings were then digitized and stored using a MS-DOS based data acquisition system (Digidata 1200 interface, Axon Instruments) and Fetchex software. Data were analyzed offline using the Mini Analysis program by Synaptosoft. Three consecutive records were averaged and the means ± SEM of the pEPSP slope (10 to 90%) were plotted versus time in the graph. Data were normalized to respective 10 min pre-tetanic controls. *n* refers to number of slices studied.

For western blots, bands were quantified using densitometric analysis and changes in the protein expression were calculated as the ratio of band of interest with the density of β- actin.

For statistical analysis, a Student's t-test was performed using GraphPad Prism software. All values are reported as mean ± SEM. The level of significance was taken as *p<0.05*.

## Results

### To determine the role of mGluRs, NMDARs and GABA_B_-Rs in LTD of pEPSPs in NS and SD rats

**Figure 1:**
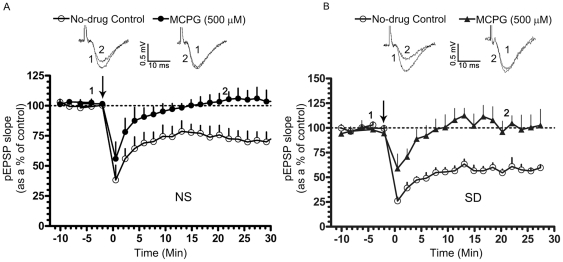
Role of mGluRs in LTD. LTD was induced using a tetanic stimulation (20-Hz for 30 s, arrow). In A and B, slices from normally-sleeping (NS) rats and sleep-deprived (SD) rats, respectively, LTD was significantly attenuated in the presence of MCPG (500 µM), a group I/II mGluR antagonist. Graph depicts the time course of LTD in no-drug controls (A, B; open circles, n = 6) and in MCPG (A, filled circles, n = 6; B, filled triangles, n = 6). Representative pEPSP traces in no-drug controls and treated conditions were taken at the indicated time points. pEPSP slopes were averaged every min and normalized to respective pre-tetanic controls.

Stimulation of the Schaffer collateral pathway, at 20-Hz for 30 s, reliably induced a depression of the pEPSP that lasted for at least 30 min. Consistent with our previous findings, LTD was significantly enhanced in SD rats [8]. Since the early phase may be a short-term depression, pEPSPs were quantitated at 10 and 20 min post-tetanus and any depression was presumed to be LTD (pEPSP slope represented as a % of the pre-tetanic control; in normally sleeping rats- 10 min post-tetanus: 74.58±9.36, n = 6; 20 min post-tetanus: 75.76±7.97, n = 6; p<0.05; Fig. 1 A; in SD rats- 10 min post-tetanus: 55.44±4.33, n = 6; 20 min post-tetanus: 54.54±3.62, n = 6; p<0.05; Fig. 1 B).

**Figure 2:**
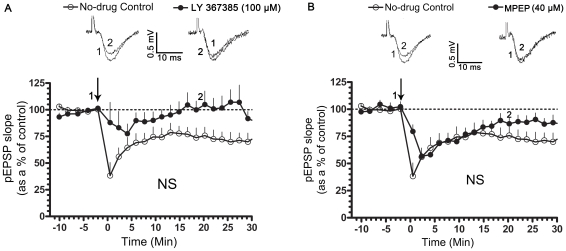
Role of mGlu1Rs and mGlu5Rs in LTD. LTD was induced using a tetanic stimulation (20-Hz for 30 s, arrow). In A and B, slices from normally-sleeping (NS), LTD was significantly attenuated in the presence of LY 367385 (100 µM), a mGlu1R antagonist or MPEP (40 µM), a mGlu5R antagonist. Graph depicts the time course of LTD in no-drug controls (A, B; open circles, n = 6), in LY 367385 (A, filled circles, n = 6) and MPEP (B, filled circles, n = 6). Representative pEPSP traces in no-drug controls and treated conditions were taken at the indicated time points. pEPSP slopes were averaged every min and normalized to respective pre-tetanic controls.

**Figure 3:**
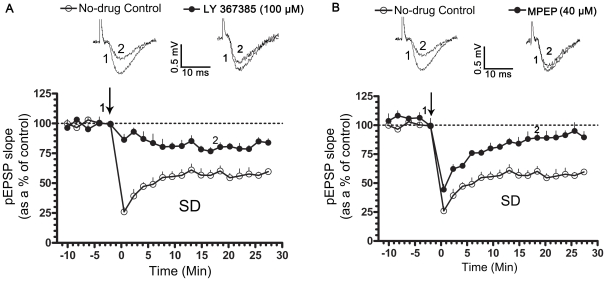
Role of mGlu1Rs and mGlu5Rs in LTD. LTD was induced using a tetanic stimulation (20-Hz for 30 s, arrow). In A and B, slices from sleep-deprived (SD) rats, LTD was suppressed in the presence of LY 367385 (100 µM), a mGlu1R antagonist or MPEP (40 µM), a mGlu5R antagonist. Graph depicts the time course of LTD in no-drug controls (A, B; open circles, n = 6), in LY 367385 (A, filled circles, n = 6) and MPEP (B, filled circles, n = 6). Representative pEPSP traces in no-drug controls and treated conditions were taken at the indicated time points. pEPSP slopes were averaged every min and normalized to respective pre-tetanic controls.

We tested the requirement for activation of mGluRs and NMDARs in the induction of 20-Hz LTD using (RS)-a-Methyl-4-carboxyphenylglycine (MCPG), a broad spectrum group I/II mGluR antagonist, (S)-(+)-α-Amino-4-carboxy-2-methylbenzeneacetic acid (LY 367385), a mGlu1R antagonist, 2-Methyl-6-(phenylethynyl)pyridine hydrochloride (MPEP), a mGlu5R antagonist [13] and APV, a competitive NMDAR antagonist, respectively. MCPG attenuated LTD in normally sleeping and SD rats, with no significant change on the baseline pEPSP (pEPSP slope represented as a % of the pre-tetanic control; in normally sleeping rats- 10 min post-tetanus: 97.24±4.61, n = 6; 20 min post-tetanus: 102.8±5.53, n = 6; p>0.05; Fig. 1A; in SD rats- 10 min post-tetanus: 108.1±11.69, n = 5; 20 min post-tetanus: 95.89±11.11, n = 5; p>0.05; Fig. 1B). Application of LY 367385 (100 µM) or MPEP (40 µM) significantly suppressed LTD in normally sleeping (pEPSP slope represented as a % of the pre-tetanic control, in no-drug controls- 20 min post-tetanus: 75.76±7.97, n = 6; p<0.05; Fig. 2A, 2B; in LY 367385- 20 min post-tetanus:105.0±12.56, n = 6; p>0.05; Fig. 2A; in MPEP- 20 min post-tetanus: 86.57±5.291, n = 6; p>0.05; Fig. 2B) and SD rats (pEPSP slope represented as a % of the pre-tetanic control- in no-drug controls- 20 min post-tetanus: 54.54±3.62, n = 6, p<0.05; Fig. 3A, 3B; in LY 367385- 20 min post-tetanus: 80.69±2.777, n = 6; p<0.05; Fig. 3A; in MPEP- 89.06±5.896, n = 6; p>0.05; Fig. 3B). In addition, LY 367385 significantly suppressed the short-term LTD in normally sleeping (pEPSP slope represented as a % of the pre-tetanic control, in no-drug controls- 38.26±12.33, n = 6, p<0.05; Fig. 2A; in LY 367385- 88.01±18.98, n = 6; p>0.05; Fig. 2A) and SD rats (pEPSP slope represented as a % of the pre-tetanic control, in no-drug controls- 26.04±3.73, n = 6; p<0.05; Fig. 3A, 3B; in LY 367385- 86.25±4.69, n = 6; p>0.05; Fig. 3A). LTD was, however, not affected in APV (pEPSP slope represented as a % of the pre-tetanic control in normally sleeping rats; 10 min post-tetanus: 75.51±1.79, n = 6; 20 min post-tetanus: 72.39±2.89, n = 6; p<0.05), indicating that the LTD induced by a 20-Hz tetanus requires the activation of mGluRs but not NMDARs.

**Figure 4:**
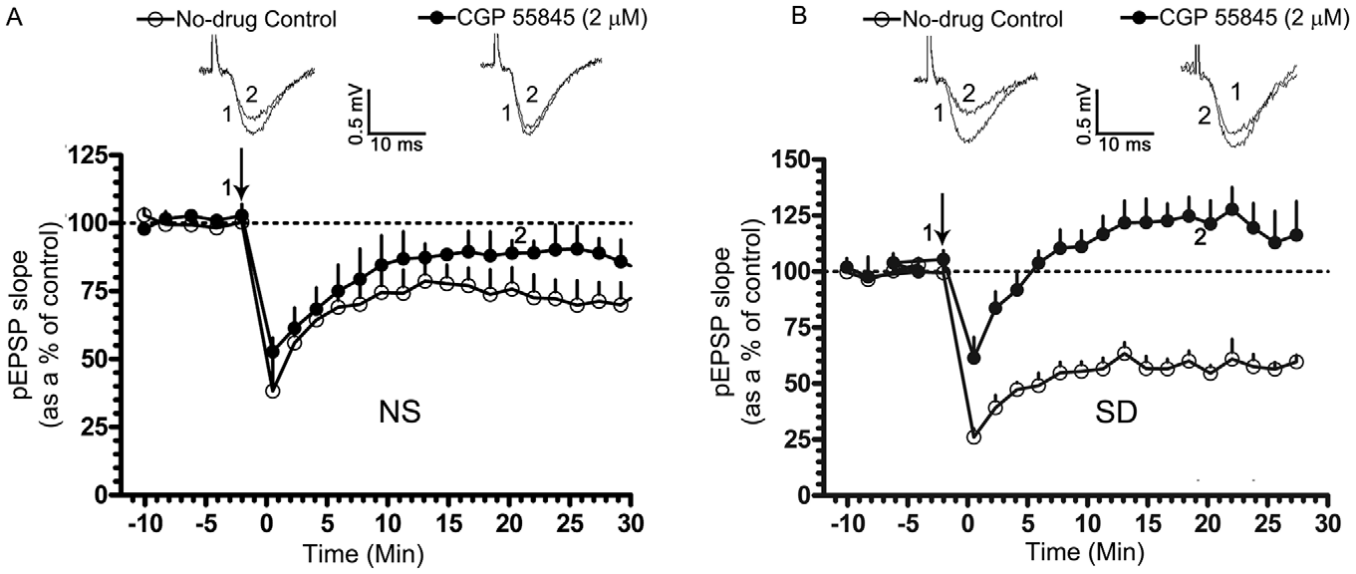
Role of GABA_B_Rs in LTD. LTD was induced using a low-frequency tetanic stimulation (LFTS, 20-Hz for 30 s, arrow). In A and B, slices from normally sleeping (NS) and sleep-deprived (SD) rats, respectively, while LTD was partially suppressed in the presence of CGP 55845 (2 µM), a GABA_B_ receptor antagonist, in NS rats, it was completely blocked in SD rats. Graph depicts the time course of LTD in no-drug controls (A, B; open circles, n = 6) and in CGP 55845 (A, B; filled circles, n = 6). Representative pEPSP traces in no-drug controls and treated conditions were taken at the indicated time points. pEPSP slopes were averaged every min and normalized to respective pre-tetanic controls.

**Figure 5:**
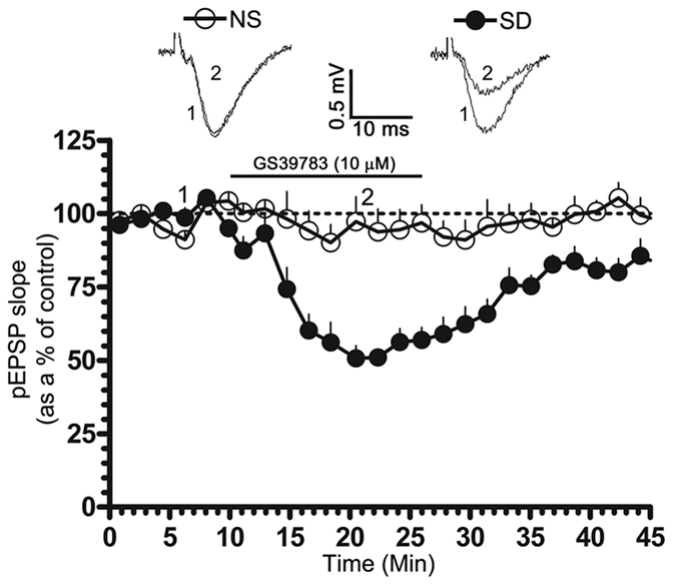
Effects of GS 39783 on pEPSPs evoked from normally sleeping (NS) and sleep-deprived (SD) rats. Bath application of GS 39783 (10 µM), selectively suppressed evoked pEPSPs in SD rats, but not in NS rats. Graph depicts the time course of evoked pEPSPs in NS (open circles, n = 5) and SD (filled circles, n = 5) conditions. Representative pEPSP traces in NS and SD conditions were taken at the indicated time points. pEPSP slopes were averaged every min and normalized to respective pre-tetanic controls.

We examined the involvement of GABA_B_ receptors in the 20-Hz-induced LTD. Bath application of CGP 55845 (2 µM), a GABA_B_ receptor antagonist, only partially blocked LTD in normally sleeping rats (pEPSP slope represented as a % of the pre-tetanic control; 10 min post-tetanus: 86.93±10.10, n = 6; 20 min post-tetanus: 89.13±4.29, n = 6; p<0.05; Fig. 4A). In SD rats, however, LTD was completely blocked in the presence of the drug (pEPSP slope represented as a % of the pre-tetanic control; 10 min post-tetanus: 116.7±8.091, n = 6; p>0.05, n = 6; 20 min post-tetanus: 121.8±9.793, n = 6; p>0.05; Fig. 4B). Moreover, application of GS-39783, a positive allosteric modulator for GABA_B_ receptors [35], significantly depressed evoked pEPSPs in SD rats (pEPSP slope represented as a % of the pre-tetanic control; 10 min post-application: 50.66±4.29, n = 5 p<0.05; Fig. 5). No change was, however, noted in normally sleeping rats (pEPSP slope represented as a % of the pre-tetanic control; 10 min post-application: 97.28±8.50, n = 5 p>0.05; Fig. 5). Since GS-39783 affects GABA_B_ receptor mediated responses only when activated, it appears that in normally sleeping rats these receptors are not fully active or not coupled to LTD induction mechanisms under a 20 Hz stimulation but get recruited in SD rats.

### The role of Ca^2+^ in 20-Hz-induced LTD of pEPSPs

**Figure 6:**
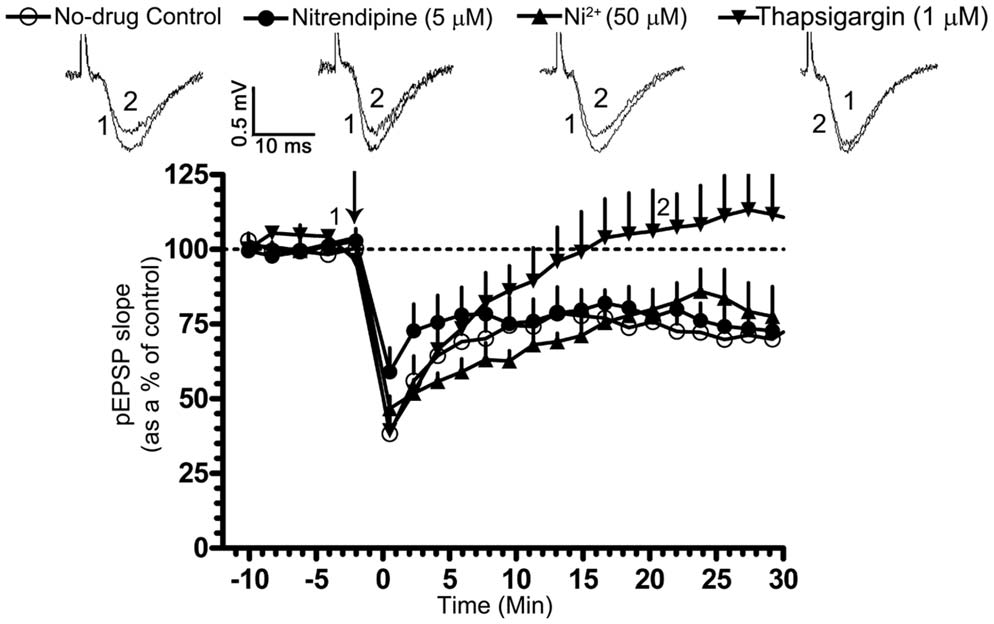
Requirement of Ca^2+^ release for LTD. LTD was induced using a low-frequency tetanic stimulation (LFTS, 20-Hz for 30 s, arrow). In slices from normally sleeping rats, LTD was blocked in the presence of Thapsigargin (1 µM), which blocks Ca^2+^-release from intracellular stores. Nitrendipine (5 µM), an L-type voltage-gated calcium channel (VGCC) antagonist or Ni^2+^ (50 µM), a T-type VGCC antagonist, however, did not affect LTD. Graph depicts the time course of LTD in no-drug controls (open circles, n = 6), in thapsigargin (filled inverted triangles, n = 6), in Nitrendipine (filled circles, n = 6) and in Ni^2+^ (filled triangles, n = 6). Representative pEPSP traces in no-drug controls and treated conditions were taken at the indicated time points. pEPSP slopes were averaged every min and normalized to respective pre-tetanic controls.

Bath application of thapsigargin (1 µM), which blocks Ca^2+^-release from intracellular stores dampened LTD induction. This effect was significant at 20 min post-tetanus (pEPSP slope represented as a % of the pre-tetanic control in normally sleeping rats; 10 min post-tetanus: 86.19±8.29, n = 6; p<0.05; 20 min post-tetanus: 106.1±13.7, n = 6; p>0.05; Fig. 6). Nitrendipine (5 µM), an L- type Ca^2+^ channel antagonist did not affect LTD induction (pEPSP slope represented as a % of the pre-tetanic control in normally sleeping rats; 10 min post-tetanus: 75.95±7.46, n = 6; 20 min post-tetanus: 79.95±8.78, n = 6; p<0.05; Fig. 6). Ni^2+^ (50 µM), which blocks T- type Ca^2+^ channels, also did not affect LTD (pEPSP slope represented as a % of the pre-tetanic control in normally sleeping rats; 10 min post-tetanus: 61.28±4.40, n = 6; 20 min post-tetanus: 79.9±4.69, n = 6; p<0.05; Fig. 6). These results indicate that Ca^2+^ release from intracellular stores, but not L- or T- type voltage-gated Ca^2+^ channels (VGCCs), are required for LTD induction.

### Expression of mGlu1αR, GABA_B_-R1 and GABA_B_-R2 in the CA1 hippocampal region of normally sleeping and SD rats

**Figure 7:**
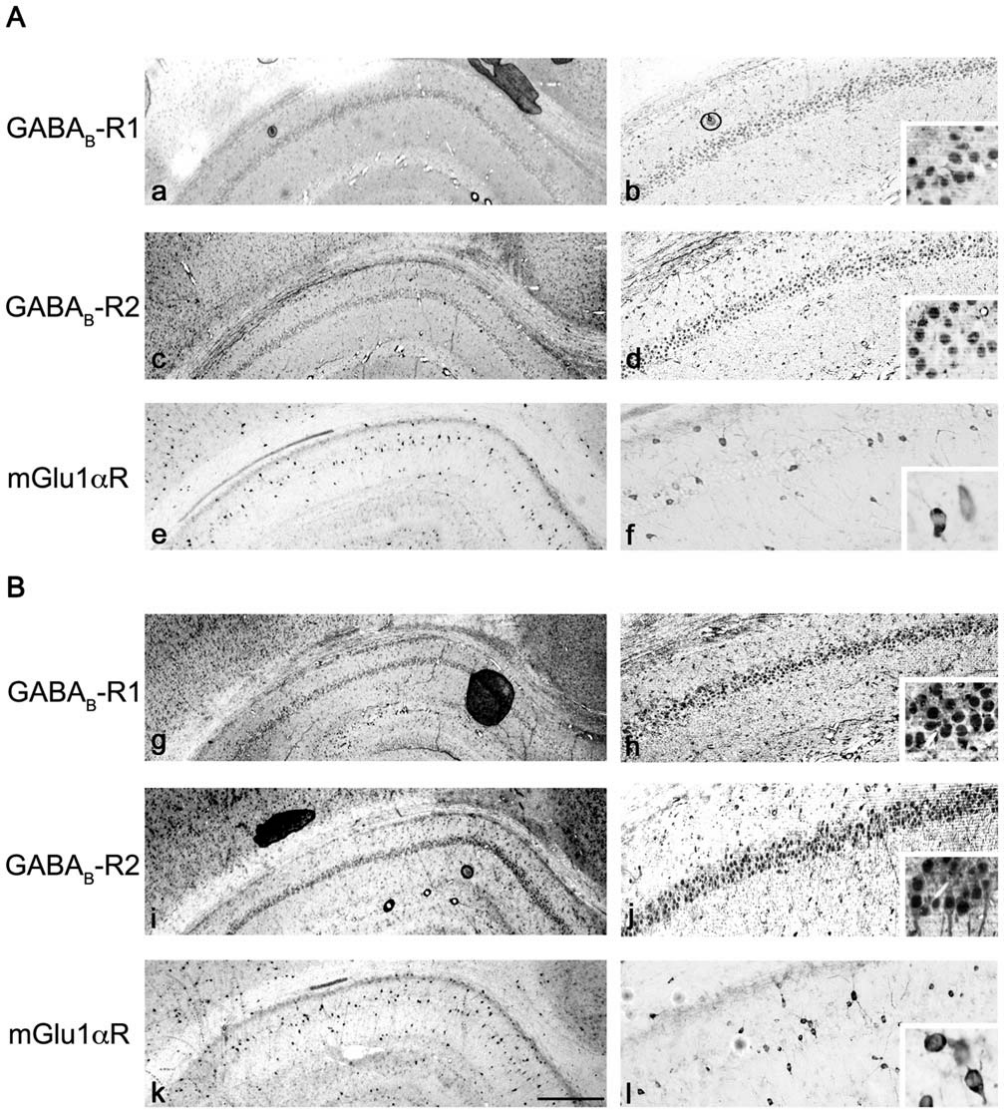
Immunohistochemical analysis of the expression of GABA_B_-R1/R2 and mGlu1αRs in normally sleeping (NS) and sleep-deprived (SD) rats. Representative photomicrographs illustrating immunohistochemical localization of GABA_B_-R1/R2 and mGlu1αR in the CA1 region of the hippocampus of NS (A) and SD (B) rats. GABA_B_-R1- like immunoreactivity was well-expressed at the neuronal perikarya and cytoplasmic protein (a, b). Following sleep-deprivation, the intensity of staining and the number of neurons immunopositive to GABA_B_-R1 increased (g, h). An enhanced immunoreactivity was also evident in the stratum radiatum (sr; b, h). For the GABA_B_-R2 subtype, a moderate to weak immunoreactivity was observed in stratum pyramidale (sp) and sr of NS rats (c, d), with a modest increase in immunoreactivity in SD rats (i, j). mGlu1αR- like immunoreactivity was strong- to moderately- expressed in isolated pyramidal and non-pyramidal neurons in the sp, sr and stratum oriens (so; e) in NS rats. Following sleep-deprivation, the intensity of staining and the number of neurons immunopositive to mGlu1α increased (k, l compared to e, f). The immunoreactivity in dendrites and axons was also enhanced. While the staining in pyramidal/non-pyramidal neuron perikarya was uniform, it appeared punctated in dendrites and axons in both NS and SD rats. Scale Bar = 160 µm for panels on left in A & B; 20 µm for panels on right in A & B; and 5 µm for inset.

Results from electrophysiological studies, therefore, indicate an involvement of mGlu- and GABA_B_- receptors in 20-Hz LTD. The differential effect of mGluR and GABA_B_-R antagonists on the time-course of LTD in normally sleeping and SD rats and the selective suppression of evoked pEPSPs in the presence of GS-39783 in SD rats, suggests that mGlu- and GABA_B_- receptors may be altered during sleep-deprivation. Since mGluRs, specifically the mGlu1αR subtype, have been previously shown to interact functionally with GABA_B_-Rs in the cerebellar parallel fiber-Purkinje cell synapses [36], we first examined if sleep-deprivation induces an alteration in the expression of mGlu1αRs and GABA_B_-Rs. A change in expression in SD rats may explain the enhancement in LTD following a 12 hour sleep-deprivation. Using peroxidase immunocytochemistry, we therefore, studied the distribution of these receptors in hippocampal brain sections from normally sleeping and SD rats (Fig. 7, a- l). In normally sleeping rats, strong GABA_B_-R1- like immunoreactivity was observed in the stratum pyramidale. Pyramidal and non-pyramidal cells in the CA1 region displayed GABA_B_-R1- like immunoreactivity at the neuronal perikarya, as well as, in intracellular compartments. In contrast, only a weak immunoreactivity was observed in the stratum radiatum (Fig. 7a, b). These findings are largely consistent with previous studies [27]. Following sleep-deprivation, however, the GABA_B_-R1- like immunoreactivity was stronger in stratum pyramidale and in the apical dendritic regions of the stratum radiatum (Fig. 7g, h). The intensity of staining and the number of neurons immunopositive to GABA_B_-R1 increased when compared to that in normally sleeping rats. Similar results were obtained for GABA_B_-R2 expression between normally sleeping and SD rats. Following sleep deprivation, the immunoreactivity in stratum pyramidale and radiatum was more pronounced in SD rats (Fig. 7i, j) in comparison to normally sleeping rats (Fig. 7c, d).

The expression of mGlu1αR also varied in SD rats. In general, in both normally sleeping and SD groups, mGlu1αR- like immunoreactivity was strong or moderately expressed in isolated pyramidal and non-pyramidal neurons in stratum pyramidale. The expression was uniform at the neuronal perikarya and in the cytoplasmic compartment. However, in axonal processes or dendrites, the immunoreactivity was mostly punctated (Fig. 7e, f). Significantly, when compared to normally sleeping rats, SD rats displayed an increase in the number of mGlu1αR positive neurons. Unlike normally sleeping rats, most pyramidal/non-pyramidal neurons in SD rats displayed a strong immunoreactivity. The mGlu1αR- like immunoreactivity at the neuronal perikarya and intracellular compartments of both cell types was more intense in stratum pyramidale and radiatum.

### Western blot analysis of mGlu1αR, GABA_B_-R1 and GABA_B_-R2 expression in the CA1 hippocampal region of normally sleeping and SD rats

**Figure 8:**
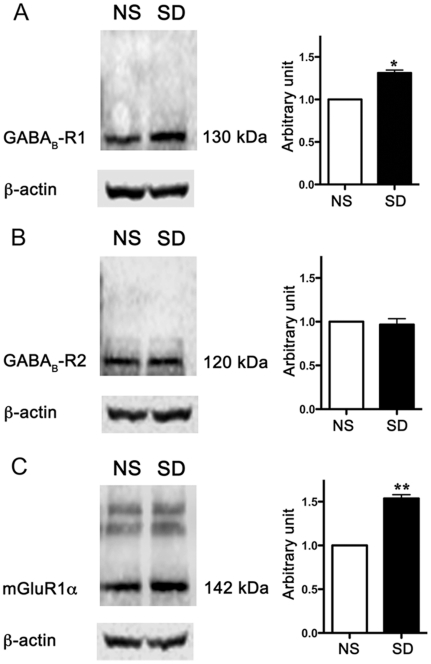
Western blot analysis of the expression of GABA_B_-R1, GABA_B_-R2 and mGlu1αR, in normally sleeping (NS) and sleep-deprived (SD) rats. Hippocampal tissue lysate from NS and SD rats were processed for western blot analysis as described in Materials and Methods section. GABA_B_-R1 (A), GABA_B_-R2 (B) and mGlu1αR (C) immunoreactivity was expressed as a single band at the expected molecular mass of ∼130 kDa, ∼120 kDa & ∼142 kDa, respectively. Histograms in representative panels describe the quantitative analysis for the receptor- immunoreactivity. A significant increase in GABA_B_-R1 and mGlu1αR protein levels was detected in SD rats with only a mild change in GABA_B_-R2 immunoreactivity. β-actin was used as the control for loading protein. * p<0.05, ** p<0.01.

Data from immunohistochemical studies therefore indicate an increase in GABA_B_-R1 and mGlu1αR immunoreactivity, with a modest change in GABA_B_-R2 expression. We further quantified these results by studying protein levels of GABA_B_-R1, GABA_B_-R2 and mGlu1αR. The hippocampal tissue lysate prepared from normally sleeping and SD rats was processed for western blot analyses, as described in material and methods. As shown in Fig. 8, GABA_B_-R1 (A), GABA_B_-R2 (B) and mGlu1αR (C) immunoreactivity was expressed as a single band at the expected molecular mass of ∼130 kDa, ∼120 kDa & ∼142 kDa, respectively. A significant increase in GABA_B_-R1 and mGlu1αR protein levels was detected in SD rats with a subtle change in GABA_B_-R2 immunoreactivity. Histograms in representative panels describe the quantitative analysis for the receptor- immunoreactivity. These findings corroborate and further strengthen our observations from immunohistochemical studies.

### Co-localization of mGlu1αR and GABA_B_-R1/R2 in normally sleeping and SD rats

It has been previously reported that GABA_B_-R1 and mGlu1Rs co-localize in cerebellum [36]. However, it is currently not known if these receptors co-localize in the CA1 hippocampal region and if sleep-deprivation causes a change in their co-expression. Therefore, in the current study, we examined if GABA_B_-R1, GABA_B_-R2 and mGlu1Rs co-localize in normally sleeping rats and compared the results with SD rats.

**Figure 9:**
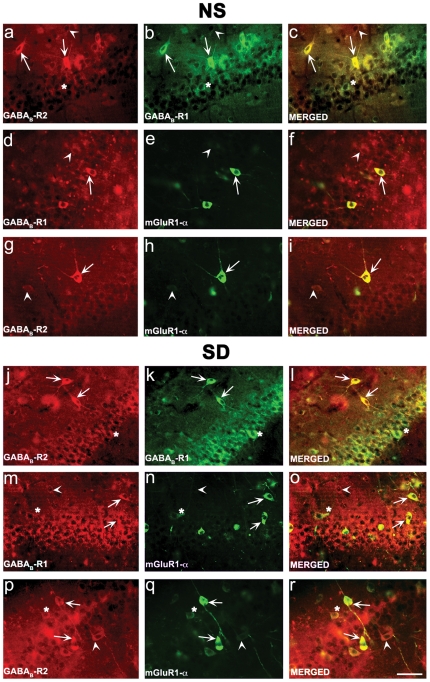
Co-localization of GABA_B_-R1/GABA_B_-R2, GABA_B_-R1/mGlu1Rα and GABA_B_-R2/mGlu1Rα in normally sleeping (NS) and sleep-deprived (SD) rats. Representative immunofluorescence photomicrographs illustrating co-localization of GABA_B_-R1/GABA_B_-R2 (top panel), GABA_B_-R1/mGlu1Rα (middle panel) and GABA_B_-R2/mGlu1Rα (bottom panel) in CA1 area of NS and SD rats. Receptors co-localization was performed as described in Material and Methods. Brain sections passing through hippocampus were stained with specific antibodies for GABA_B_-R1, GABA_B_-R2 and mGlu1Rα. Red and green fluorescence indicate individual receptor types while yellow (merged) shows co-localization. Note the specific increase in receptor immunoreactivity and co-localization in SD rats in comparison to normally sleeping rats. Arrows in representative panels indicate co-localization, arrow-heads indicate neuronal population devoid of co-localization, whereas, asterisks indicate mild co-localization. Scale Bar = 10 µm.

To investigate whether GABA_B_-R1/GABA_B_-R2 or GABA_B_-R1/mGlu1αR and GABA_B_-R2/mGlu1αR co-express in the CA1 region of the hippocampus, double-labelled immunofluorescence co-localization was performed. In agreement with previous studies [27], a significant overlap in distribution of GABA_B_-R1 & R2- like immunoreactivity was observed in the CA1 pyramidal neurons between normally sleeping (Fig. 9, a–c) and SD rats (Fig. 9, j–l). Much of the co-localization is confined to the neuronal perikarya. Importantly, as illustrated, GABA_B_-R1 expression was enhanced in SD rats (Fig. 9k) in comparison to normally sleeping rats (Fig. 9b) without any discernible changes in co-localization (Fig. 9l, c).

We further extended our study to determine the co-localization between GABA_B_-R1/mGlu1αR in normally sleeping- (Fig. 9, d–f) and SD- (Fig. 9, m–o) rats. In normally sleeping rats, co-localization between GABA_B_-R1 and mGlu1αR was mainly restricted to stratum pyramidale and confined at the pyramidal/non-pyramidal cell perikarya. In addition, discrete neuronal population displayed punctated co-localization in the dendrites. However, in SD rats, the immunoreactivity for both GABA_B_-R1 and mGlu1αRs was significantly enhanced in stratum pyramidale. The number of pyramidal/non-pyramidal neurons exhibiting co-localization for mGlu1αR and GABA_B_-R1 clearly increased. Co-localization, as in the normally sleeping rats, was, however, mainly restricted to the neuronal perikarya, although some neurons displayed an increased co-localization in the dendrites.

As illustrated in Fig. 9, the GABA_B_-R2/mGlu1αR displayed strong co-localization in hippocampal CA1 region in SD (Fig. 9, p–r) rats when compared with normally sleeping (Fig. 9, g–i) rats. In stratum pyramidale, the number of individual pyramidal/non-pyramidal neurons co-expressing GABA_B_-R2 and mGlu1αR increased in SD- when compared to normally sleeping- rats. This increase was not only restricted to the neuronal perikarya, but also was clearly evident in the dendrites.

### GABA_B_-R1 and GABA_B_-R2 receptors are expressed in mGlu1α receptor immunoprecipitate in normally sleeping and SD rats

**Figure 10:**
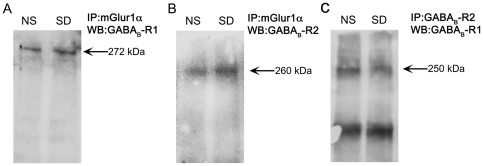
Heterodimerization between GABA_B_-R1/mGlu1αR, GABA_B_-R2/mGlu1αR and GABA_B_-R1/R2 in normally sleeping (NS) and sleep-deprived (SD) rats. Co-immunoprecipitation analysis illustrating the heterodimerization between, GABA_B_-R1/mGlu1αR (A), GABA_B_-R2/mGlu1αR (B) and GABA_B_-R1/R2 (C), in hippocampal tissue lysate from NS and SD rats. As described in the Materials and Methods section, the tissue lysate was immunoprecipitated with mGlu1αR or GABA_B_-R2 specific antibodies. The mGlu1αR- or GABA_B_-R2- immunoprecipitate was then fractionated on a 7% SDS gel and probed with anti-GABA_B_-R1/R2 or GABA_B_-R1 antibodies, respectively. Note the formation of GABA_B_-R1/mGlu1αR (A), GABA_B_-R2/mGlu1αR (B) and GABA_B_-R1/R2 heterodimers at the expected sizes of ∼272, ∼260 and ∼250 kDa in NS and SD rats. While the complex-formation between GABA_B_-R1/mGlu1αR and GABA_B_-R2/mGlu1αR is clearly enhanced in SD rats, GABA_B_-R1/R2 heterodimerization is reduced.

Our immunocytochemical, western blot and co-localization analysis of GABA_B_- and mGlu1α- receptors suggest that these receptors may function as heterodimers in hippocampus. Whether GABA_B_- and mGlu1α- receptors form a complex and functionally interact with each other in cerebellum is currently disputed [37] and not known in the hippocampus. Therefore, we performed co-immunoprecipitation experiments to study the complex formation between GABA_B_-R1/mGlu1αR and GABA_B_-R2/mGlu1αR, as well as, between GABA_B_-R1/GABA_B_-R2. As shown in Fig. 10A, GABA_B_-R1 expression was detected at the expected size of ∼272 kDa indicating a possible heteromeric complex in mGlu1αR immunoprecipitate. Similarly, a complex formation was also observed between mGlu1αR and GABA_B_-R2 at the expected molecular weight of ∼260 kDa (Fig. 10B). We further extended our study to determine whether GABA_B_-R1 forms heterodimers with GABA_B_-R2. As expected, in Fig. 10C, the immunoprecipitate of GABA_B_-R2, when probed with GABA_B_-R1 antibody, displayed a band at ∼250 kDa. The GABA_B_-R1/R2 dimerization is consistent with several previous studies in different brain regions [24], [25], [26]. In conclusion, these results indicate that GABA_B_-R1/mGlu1αR and GABA_B_-R2/mGlu1αR dimerize in hippocampus, the extent of which is clearly enhanced in SD rats (Fig. 10A, B). GABA_B_-R1/R2 heterodimerization, however, is decreased in SD rats (Fig. 10C). Evidence in literature suggests that GABA_B_-R1 and R2 may independently interact with other structurally homologous partners, such as, mGluRs, to surface express and for function [38]. For instance, co-expression of GABA_B_-R1 with mGlu4Rs in cell lines seems to aid the surface expression of GABA_B_-R1s or GABA_B_-R2s, independent of the GABA_B_-R1/R2 heterodimerization [39]. Therefore, it is possible that the decrease in GABA_B_-R1/R2 dimerization and the formation of mGlu1αR-GABA_B_-R1/R2 heterodimers, observed in our study, are related events. Although speculative, the association of mGluRs with GABA_B_-Rs may hence be sufficient to overcome the ER-retention signal for GABA_B_-R1. These aspects need further investigation.

## Discussion

LTD of the pEPSP is enhanced in SD rats. This observation is consistent with our previous findings [8]. Results from our present study indicate that 20-Hz LTD requires the activation of mGluRs and a release of Ca^2+^ from intracellular stores. Among mGluRs, there are differences: while mGlu1Rs seem to be involved in both short-term depression and LTD, mGlu5Rs appear to participate mainly in LTD. These differences are interesting and further investigation is needed to understand their functional significance. NMDARs and L- or T- type VGCCs, proposed in literature to be involved in other forms of LTD, were not required for a 20 Hz-induced LTD. When GABA_B_-Rs are blocked, LTD is diminished in normally sleeping rats but absent in SD animals. In addition, GS-39783, a positive allosteric modulator for GABA_B_-Rs, depresses pEPSPs specifically in SD rats and not in normally sleeping animals. While this is a very interesting result, currently, we don't have a clear explanation for it. Perhaps, sleep deprivation facilitates GABA_B_-R linkage to the LTD phenomenon. It is also possible that the relevant GABA_B_-Rs lay dormant and become functional with sleep deprivation. Such speculations await further investigation. Taken together, these results not only indicate a role for mGluRs and GABA_B_-Rs in LTD, but also suggest receptor specific changes during sleep-deprivation. McDermott et al. (2003) [32] failed to see an enhancement of LTD in SD rats using a 1 Hz conditioning stimulation protocol and while blocking GABA-ergic transmission. Their LTD was thought to involve NMDA receptor activation. This discrepancy could be related to differences in the experimental protocols and conditioning stimulations used. The findings further strengthen the thinking that LTD induced by distinct protocols vary in their mechanisms [40].

Using a combination of immunohistochemical, co-localization and co-immunoprecipitation techniques, we further examined if the change in mGlu1αR and GABA_B_-R function in SD rats could be related to alterations in the expression pattern of mGlu1αR, GABA_B_-R1 and GABA_B_-R2 and/or in the dimerization of these receptors. Our results indeed indicate that alterations do occur in mGlu1αRs and GABA_B_-Rs following sleep-deprivation. While mGlu1αRs and GABA_B_-Rs seem to co-operatively modulate LTD, their independent effect on LTD cannot be ruled out at this stage. Because of the complex arrangement of these receptors (presynaptic, postsynaptic, interneurons, pyramidal neurons, etc.), as mentioned elsewhere in the discussion, the intricate mechanisms can be studied, only after examining the precise locus of change. Furthermore, the role of other mGluR subtypes in LTD, remains to be established. The lack of mGluR subtype selective and potent antagonists is currently hampering the assessment of individual mGluR subtypes in LTD [41].

Sleep in humans is composed of rapid eye movement sleep (REMS) and slow wave sleep (SWS, stages I–IV), which alternate every 90 min until natural awakening [42]. In addition, memories are broadly classified into declarative and non-declarative subtypes [43]. While REMS and SWS seem to preferentially affect different forms of memory [44], we employed a total sleep deprivation protocol in the present study. This was done in-order to replicate events such as, prolonged warfare, sudden change in shift work, etc., where an individual is more likely to lose the entire sleep for a short duration. At a later stage, however, it is important to examine the effect of REMS and SWS deprivation to better understand the complex mechanisms involved in LTD and its enhancement in SD rats. The finding that the 20-Hz LTD is sensitive to activation of both mGluRs and GABA_B_-Rs is not surprising. Subcellularly, in addition to their somatic and extrasynaptic localization [45], [46], GABA_B_-Rs and mGlu1αRs have been found to be present, on the periphery of the postsynaptic densities of asymmetric synapses, in GABAergic and glutamatergic axon terminals, in hippocampus, cerebellar cortex, and elsewhere in the CNS [27], [28], [47]. Consistent with these findings, our present data indicating a co-localization between mGlu1αR and GABA_B_-R1/R2 in pyramidal/non-pyramidal cells also support an overlapping distribution for mGlu1αRs and GABA_B_-Rs in the CA1 region of hippocampus. However, to activate perisynaptic/extrasynaptic- mGlu1αR or GABA_B_-R, intense synaptic activity is thought to be necessary [27], [48]. The 20-Hz repetitive stimulation employed in this study, is powerful enough to not only induce homo- and hetero- synaptic depressions [49], but also unmask the otherwise latent excitatory synaptic connections in hippocampal neurons [50]. Stimulating afferent fibers at this rate may, therefore, be strong enough to simultaneously activate both GABA_B_-Rs and mGlu1αRs and provoke an interaction in a manner that is sufficient to induce and modulate LTD. Alternatively, previous studies have shown that, in a functionally active heteromeric complex, the activation of a single protomer is sufficient to modulate its counterpart to trigger a physiological response [22]. Hence, in a scenario where mGlu1αRs and GABA_B_-Rs form complexes, activation of one receptor may not only further stimulate the formation of a heteromeric complex, but also enhance/decrease the effect of the other. In fact, a functional cross-talk/dimerization between mGlu1αR and GABA_B_-R in cerebellar parallel fiber-Purkinje cell synapses seems to lead to a synergistic enhancement in the effect of mGlu1αR [29]. Conversely, mGlu1R antagonists seem to enhance GABAergic neurotransmission, thereby significantly reducing post-ischemic neuronal damage and epileptiform activity [51]. In either scenario, it is hence possible that mGlu1αRs and GABA_B_-Rs act in concert to affect LTD of the pEPSP, and alter it during sleep-deprivation. Therefore, exactly how GABA_B_-R1/R2-mGlu1αRs dimerize to influence 20-Hz LTD and its enhancement in SD rats must be further investigated. Also, selective synergistic functional interactions between mGlu1αR and GABA_B_-R agonists following sleep-deprivation needs to be determined. If a synergy, as predicted, exists, this would have significant implications for GABA_B_-R and mGluR pharmacology in the CNS.

GPCRs exist as homo-, hetero- and, in higher order, as oligo- mers with a variety of proteins. Functionally, GPCR dimerization seems to influence, receptor surface expression [52], sensitivity of receptors to endogenous ligands [53], [54], signal transduction [22] and receptor internalization [55], [56]. Interestingly, although each of these aspects can affect the induction of LTP and LTD in the CNS, the role of GPCR dimerization on synaptic plasticity remains largely unexplored. mGlu1αRs and GABA_B_-Rs, in addition to interacting with each other, can also independently form stable heteromeric complexes with other receptors, such as, calcium sensing receptors [57] and a variety of cytoskeletal, scaffolding and signalling proteins like MUPP1, Homer and Shank [58], [59]. In a majority of cases, specific domains on the carboxyl-terminal tails of GPCRs seem to be important for an interaction to take place [26], [60], [61]. However, the GABA_B_-R-induced amplification of mGlu1R responses is intact even when it is co-expressed with mGlu1βR, a short splice variant with a much smaller carboxyl-terminal tail [37]. Further, disulphide bridges between cysteine residues of the extracellular N-terminal seem to be critical for homodimerization of mGlu1Rs [62]. These data indicate that GPCR dimerization is a complex process, unique to each receptor pair. Therefore, it is important to elucidate the mechanisms behind GABA_B_-mGlu1α receptor interaction, and any possible alterations following sleep-deprivation.

While modulation of LTD via receptor heterodimerization is a novel concept, the current study does not exclude the possibility of GABA_B_-Rs and mGlu1αRs independently affecting the 20-Hz LTD. GABA_B_-Rs and mGluRs modulate neuronal excitability and synaptic plasticity through actions on pre- and/or post- synaptic targets. Activation of mGluRs (mainly, group II & II) & GABA_B_-Rs expressed on glutamatergic- and GABA-ergic- axon terminals regulate neurotransmitter release by inhibiting presynaptic VGCCs and/or by interfering directly with the transmitter release machinery [63], [64]. Stimulation of mGluRs (mainly, group I), localized on postsynaptic neurons, depolarize them through actions on various Ca^2+^, K^+^ and other non-specific cationic conductances [64], [65]. Conversely, postsynaptic GABA_B_-Rs, when activated, cause a G-protein-mediated alteration in K^+^/Ca^2+^ conductance, leading to either a shunting or hyperpolarizing inhibition of the target neuron [66]. However, since GABA_B_-Rs and mGlu1αRs are expressed on dendrites, soma and axons of both GABA-ergic and glutamatergic neurons, the assessment of the net effect of activation of these receptors on the plasticity of pEPSPs is rather complicated. One must therefore, first establish the locus of change for mediating 20-Hz LTD, whether it is at the level of pyramidal neurons or GABA-ergic interneurons of the stratum radiatum or both, in future studies.

The 20-Hz LTD is dependent on Ca^2+^-release from intracellular stores. This observation is consistent with the current view that activation of mGluRs stimulates Ca^2+^-release from intracellular stores [67]. An enhanced LTD is observed during aging, and is linked to impaired learning, consolidation and rapid forgetting in those animals [12]. Although, the mechanisms involved in this LTD are poorly understood, Ca^2+^-release from intracellular stores seems to be critical [68]. Since SD rats also exhibit an elevated LTD [8] and similar behavioural characteristics, the role of Ca^2+^-release from intracellular stores in LTD is interesting.

### Conclusion

In conclusion, sleep-deprivation has become increasingly common owing to lifestyle changes, warfare and pathological conditions, such as, Alzheimer's disease, sleep apnea etc. Despite this, our knowledge of how sleep-deprivation affects synaptic transmission and plasticity remains very limited. One of the significant findings of the current study is that sleep-deprivation for as little as 12 hours alters the expression of GABA_B_-Rs, mGlu1αRs and LTD of the pEPSP. Chronic sleep-deprivation may therefore have more profound effects on synaptic plasticity, the balance between excitatory and inhibitory systems and the basic functioning of the CNS. A careful examination of these aspects will aid in the identification of novel targets and design of better drugs to combat problems like sleep-deprivation induced amnesia.
